# Influences of Boundary-Spanning Leadership on Job Performance: A Moderated Mediating Role of Job Crafting and Positive Psychological Capital

**DOI:** 10.3390/ijerph191912725

**Published:** 2022-10-05

**Authors:** Rukuan Xue, Hyung Rok Woo

**Affiliations:** Department of Business Administration, Mokpo National University, Muan-gun 58554, Korea

**Keywords:** boundary-spanning leadership, positive psychological capital, job crafting, job performance

## Abstract

Due to the pursuit of a flexible organization, the interest in internal as well as external boundary management has increased as a key to achieve high performance. This study identified boundary-spanning leadership (BSL) as a critical factor enhancing job crafting and job performance of subordinates. By examining the mediating effect of job crafting and the moderating effect of positive psychological capital (PsyCap), the authors aimed to present the effects of BSL assimilating job resources, such as external information and knowledge into organization. Data were collected from 238 workers of 11 companies using questionnaires in South Korea to test the moderated mediation model. The results of regression analysis using LISREL and SPSS, revealed that the manager’s BSL provoked job crafting, which in turn achieved job performance of subordinates. Moreover, PsyCap positively moderated the effect of BSL on job crafting of subordinates. The findings suggested, therefore, in order to maximize the positive effects of BSL in an organization, it should also be necessary to understand and boost PsyCap along with enhancing job crafting.

## 1. Introduction

A dynamic business environment and economic globalization are changing the importance and urgency of the resources required to achieve organizational performance. In practice, acquiring and disseminating useful knowledge from inside and outside the organization is considered critical as knowledge-based work increases [1,2]. In response to these environmental changes, “external activities”, which cross the boundary of the organization and secure new resources by interacting with the external environment, are receiving warranted attention [3,4]. Furthermore, the need has long been felt for a manager’s activities to adapt to and interact with the external environment [5]. The manager is described as “linking pin” or “gatekeeper”, highlighting the external oriented role as a bridge facilitating mutual resource exchange with outsiders [6,7].

Boundary-spanning leadership (BSL) refers to behavior that contributes to the achievement of goals by internal subordinates by exploring and acquiring the necessary resources and information through communication with the outside and disseminating them to the subordinates internally [8,9]. Considering that leadership is the process of influencing subordinates to achieve goals, the theoretical and practical value of BSL is crucial, since it provides the necessary resources to subordinates while simultaneously managing the external environment and creating organizational performance [10]. Despite the significant accumulation of academic research results regarding leadership, the verification of how BSL affects the attitude and performance of subordinates, and thus the effectiveness of the organization, remains insufficient.

To fill the above research gap, this study focused on job crafting and the positive psychological capital (PsyCap) of subordinates. In a recent research on organizational behavior of workers, the effect of job crafting and PsyCap on performance has attracted attention [11,12]. First, this study considered job crafting could mediate the relationship between a manager’s BSL and subordinates’ job performance. Job crafting is a voluntary change effected to balance job demand and job resources and makes it possible to achieve performance under environmental challenges, e.g., as seen in [13,14]. We assumed that this positive impact of job crafting will come into play even in the face of BSL creating a resource-rich work environment, which increases the flexibility of external job resources [15]. Next, this study identified the moderating role of PsyCap. PsyCap is based on positive psychology and focuses on understanding and developing human strengths. It refers to the complex positive psychological state of an individual needed to perform well on a given task and achieve successful results [16]. By developing individual strengths, PsyCap has a significant impact on motivational processes and job performance [17,18]. We examined whether PsyCap is a conditional factor influencing the dynamics among BSL, job crafting, and job performance. The results and implications of this study is to contribute to a comprehensive understanding of the efficacy of BSL.

## 2. Theoretical Background

### 2.1. Boundary-Spanning Leadership (BSL)

Managers’ boundary-spanning behavior has recently been emphasized as a more important competency in line with the advancement of industrial technology and rapid changes in knowledge-intensive technologies [7,19]. This is because the need has increased for a manager to take the lead through boundary-spanning behavior and effectively manage the flow of resources, information, and knowledge that crosses existing boundaries while efforts to secure a competitive advantage by creating performance based on innovative ideas of subordinates are also increasing [20]. BSL is a manager’s action of interacting with major parties in the external environment by crossing the boundary of the organization (team), protecting the internal boundary from external uncertainties, and strengthening solidarity [21,22,23].

Ernst and Yip [24] presented four effective strategies for managing the social boundaries of groups within an organization. The first is suspending, which creates a third space for the formation of human relationships among individual team members by developing a communal cafeteria or shelter for all employees. The second is a reframing that forms a sense of solidarity by giving a higher-level common identity that can encompass team members with diverse characteristics. The third is nesting, in which individual groups share a high-level work goal which systematically promotes interaction. Lastly, weaving is a strategy that interdependently crosses social and organizational identities. It is intended to bridge the diversity gap by interconnecting heterogeneous social identities across organizational roles and positions.

Developing these four strategies [24], recent research, e.g., [6,25], has presented six practices of BSL: buffering, reflecting, connecting, mobilizing, weaving, and transforming. They further presented three strategies, each containing two practices. Buffering and reflecting correspond to the “strategy to manage boundaries”, connecting and mobilizing correspond to the “strategy to forge common ground”, and weaving and transforming correspond to the “strategy to discover new frontiers”. The authors claim that BSL can achieve a nexus effect through these representative behaviors.

Marrone, Ferraro, and Huston [19] recently explained the manager’s boundary-spanning behavior based on existing studies, e.g., [2,21] related to boundary management as a broad category. They divide the behavior into two dimensions: boundary-loosening and boundary-tightening. Boundary-loosening activities include activities connecting with key outsiders, scouting external information, and persuading others to support group decisions. Boundary-tightening activities are composed of boundary-buffering and boundary-reinforcement activities. Boundary-buffering activities prevent the negative impact of influences, such as uncertainties and threatening situations from the outside, and simultaneously serve to protect useful internal resources from leaking outside the organization. Boundary-reinforcement activities refer to a behavior that focuses on the work commitment and identity reinforcement of internal subordinates rather than focusing outside of the organization.

The various sub-elements and concepts of BSL were examined by exploring the aforementioned previous studies. For boundary management to be successful, the inflow and dissemination of useful external information, collaboration with key stakeholders, and communication with other departments must take place smoothly. The manager’s work ability and social relationship is a factor enabling the group’s boundary-spanning behavior to be more effective [19]. However, there is a lack of empirical studies on why and how BSL affects subordinates. In addition, systematic research on BSL has not been conducted as existing leadership studies focus on dynamics within the boundary, and, thus, there is a limitation to solving practical issues. Accordingly, this study conducts an empirical verification of the influence of BSL, which supplements the deficiencies of these previous studies.

### 2.2. Job Crafting

Job crafting refers to “the physical and cognitive changes individuals make in the task and relational boundaries of their work” [26,27]. It is a proactive behavior that actively changes the identity, meaning, and scope of work [28]. Wrzesniewski and Dutton [29] explain job crafting as a concept that includes not only the organizational subordinates’ self-directed improvement of job characteristics but also a change of perception that re-discovers the meaning of the job. They presented task crafting, relational crafting, and cognitive crafting as sub-factors of job crafting.

Task crafting is an activity that improves and coordinates the number, scale, and type of tasks from the prescribed official job. For example, to finish a project quickly, an engineer could employ communicating with others. In this way, they can not only work as scientific engineers but also play a role as a guardian to protect and complete projects quickly [29,30]. It can be seen as reshaping job’s boundaries [31] because they change the approach or scope of work. Relational crafting is changing the degree of interaction with others at work. This includes trying to make friends with colleagues who have similar interests and skills at work, or when a marketer has a closer relationship with a colleague in sales team, who understands and helps them better [29,30]. In the process of carrying out the task, the frequency of interaction with stakeholders is changed, so it can be seen as reshaping relational boundaries [31]. Cognitive crafting is an activity in which organizational subordinates give meaning to their jobs and recognize the higher value of the job through reinterpretation. This is the case when hospital cleaners recognize their work as contributing to the patient’s recovery as a member of the treatment team, not just cleaning [29,30]. Since it changes the perception of the significance and goal of the job, it can be viewed as reshaping cognitive task boundaries [31].

European studies, on the other hand, consider job crafting as an activity to resolve the imbalance between job demand and job resources. In the job demands–resources model (JD-R), job demand means the emotional and cognitive effort required by subordinates for the job, and job resources means various resources and supports that subordinates can use to perform their jobs [32,33]. Based on this model, Tims, Bakker, and Derks [30] presented three behaviors that increase challenging job demands, structural, or social job resources, and one behavior that reduces hindering job demand as sub-behaviors of job crafting.

Although job crafting is conducted when an individual feels that a change is necessary in their job, enabling meaningful work experiences [34], it may be influenced by the people or the work group that the individual frequently communicates with for work-related issues [35]. A manager or leader is a prime example of this. A manager is an important influencer in the work environment, providing various tangible and intangible resources and motivating individuals to take the initiative [27,36]. For examples, job crafting has a significant relationship with empowering leadership [37,38], ambidextrous leadership [39], paradoxical leadership [40], or transformational leadership [41].

This study examines the relationship between BSL and job crafting. Numerous researchers, e.g., [5,21,42], have emphasized the role of knowledge transfer and information exchange in BSL. The manager’s act of providing a wide range of information and knowledge necessary for work makes subordinates feel they are receiving the necessary assistance to carry out their work. This influences job crafting by determining that individual growth and development can take place by utilizing the abundance of resources. Accordingly, it can be deduced that the increase in information and knowledge through BSL will have a positive relationship with the job crafting of subordinates.

### 2.3. Job Performance

Job performance is considered an important outcome in the field of organizational psychology [43]. It is as a construct that indicates to what degree the employee’s job has been successfully accomplished to contribute to organizational goal [44]. However, it is never easy to define job performance because the term includes concepts with multiple perspectives [45], such as input-to-output ratio, promotion, creativity, social influence, in-role performance, adaptability to related environments, etc. There is a consensus that it is not reasonable to understand and evaluate job performance simply based on the economic aspects.

Job performance has been studied through various dimensions according to the research purpose. Rotundo and Sackett [46] classified it into three domains: task performance, contextual performance, and counterproductive work behavior. Task performance refers to the proficiency that performed the duties required by his or her job. The second one is contextual performance, which is defined as a set of behaviors that support the social and psychological developments to bring value to the workplace. This includes extra-duty behaviors, such as enthusiasm, proactiveness, or initiative. Counterproductive work behavior is defined as negative behaviors that undermine the well-being of the organization, such as complaining, absenteeism, or tardiness. Many researchers, e.g., [40,47], when dealing with leadership or job crafting have applied overall job performance rooted in the concept of task performance, the first domain. In the same way, this study also considered job performance as all behaviors that are associated with the goals of employee’s job.

Previous studies claiming the direct effect of BSL on the job performance of subordinates argue that job performance can be improved by making more objective decisions as information and knowledge can be readily acquired and utilized from outside the boundary [48,49]. However, these studies only address the direct relationship with the performance variable; there is a lack of studies describing the specific mechanism.

Lyons [50] states that the recognition of receiving opportunities and information in advance is important in making the decision to change an individual’s job. According to this claim, a high level of flexibility in the use of various resources supported by a manager’s BSL will help individuals adapt to the skills and needs required to perform their jobs and will play a role in changing work to suit individual preferences. If subordinates receive suggestions and support from a manager engaged in BSL, they will gain enthusiasm for their jobs, and their job performance will improve through job crafting.

The conservation of resources theory [51,52] argues that acquiring ample resources is associated with more autonomous and flexible control of the information and of resources allocated to job performance and that an individual plays more roles to build new resources by reinvesting job resources. From this perspective, the BSL of a manager plays an important role in predicting the job performance of subordinates as an actor who can regulate the flow of resources and information through connections outside of the organization.

The rationale for the positive relationship between job crafting and job performance of subordinates can be found in the JD-R model. Through job crafting, which proactively changes the job to balance the work situation, the increase in job resources, challenging job demands, and the decrease in disruptive job demand play a major motivating role in promoting job performance [53]. According to this claim, individual job performance improves as the job resources increase, and, thus, the job crafting of subordinates created by BSL is highly likely to lead to improving job performance.

BSL seeks and procures resources necessary for subordinates to work with external actors and aims to spread them to subordinates [22,23]. These manager’s behaviors will help subordinates to obtain more resources and information, which is highly likely to have a positive effect on job performance and job crafting of the subordinates. This study established the hypothetical relationship between BSL of managers and the job performance of subordinates through the mediating role of job crafting.

### 2.4. Positive Psychological Capital (PsyCap)

PsyCap is a concept which began with research in the field of positive psychology and is actively accepted in mental health research. While traditional psychology is overly focused on the negative aspects of humans, positive psychology aims to contribute to a more comprehensive understanding of human behaviors by giving attention to strength [17]. It embraces the need for a more balanced approach that considers both the positive and the negative based on the concept that individual positive experience and attitudes are likely to affect not only personal life but also work life.

Along with intellectual capital, social capital, and cultural capital, PsyCap is recognized as the capital necessary for the sustainable competitive advantage of individuals and organizations [16]. PsyCap is a positive psychological trait of an individual that contributes to goal achievement and improvement in performance—that is, human psychological factors function as capital when they are positively converted and expressed as organizational effectiveness or performance [16,54,55]. The specific components of PsyCap are self-efficacy, which is the confidence needed to succeed in challenging tasks [56]; optimism, which is a positive attitude toward present and future success [57]; hope, defined as the desire and perseverance to achieve the goal [58]; and resilience, which recovers one to their original state after suffering frustration and when faced with adversity [59]. PsyCap offers a robust explanation of individual attitudes and behaviors through and understanding of self-efficacy, optimism, hope, and resilience as an integrated resource [60,61].

On the other hand, Seligman [57], known as the founder of positive psychology, emphasized understanding as an integrated psychological state in which positivity toward success and achievement is internalized. Recently, Bouckenooghe et al. [62] divided PsyCap into two resources and examines its discriminatory effects. The authors suggested, based on the conservation of resources theory, that the PsyCap is different combinations of gain-oriented resources and loss-oriented resources. The former that encourages new energy construction is related to self-efficacy, hope, and optimism. Whereas the latter helps maintain the status quo by recovering from unfavorable or stressful events and is related to resilience.

This study considered the moderating effect of PsyCap on the relationship between BSL and job crafting. An individual needs ability and persistence for job crafting [26,34]. The more confident the individual is in their abilities and the more the individual believes that they can achieve goals, the more the individual will continue to strive for job crafting. In the JD-R model, which is the theoretical basis of job crafting, individual characteristics can play the role of resources, thereby mitigating negative factors induced by job demand [53].

The positive effect of PsyCap has been demonstrated in various fields, e.g., see [16,18,60]. A higher level of PsyCap is associated with a higher level of self-esteem and self-congruence with a goal, producing desirable results in terms of motivation, job performance, and job satisfaction. Since people with a high level of PsyCap think they can solve problems given to them, they have the will to solve problems and deploy active solutions [63]. When faced with failure or adversity, people with PsyCap respond more positively because they have more internal locus of control and more positive expectations of outcomes [12]. In addition, even when an individual is confronted with a variety of demands in their job, PsyCap allows the individual to act more flexibly and adaptively.

**Figure 1:**
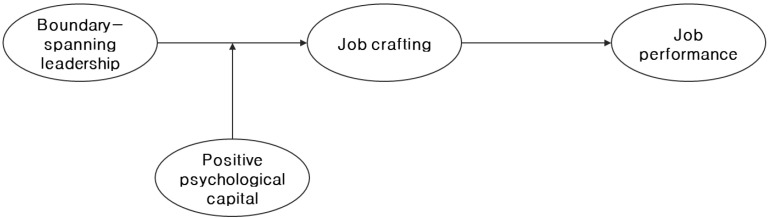
Conceptual research model.

This function of PsyCap strengthens the relationship in which the job crafting of subordinates is triggered by BSL. Subordinates with a high level of PsyCap actively responds to given demands even if they experience job impairment [18]. BSL may function as a burden for subordinates as it requires them to carry out extra-role behavior, which has not been previously performed, but PsyCap provides subordinates with the motivation to overcome these psychological pressures and adapt in a healthy and productive way. Since subordinates with a high level of PsyCap believe that they can solve the problem, they take a more active role in the process of setting their own work goals and achieving them and do not give up but look for an appropriate solution when faced with obstacles [54,64]. Based on the above discussion, this study established the following research model (see Figure 1).

## 3. Materials and Methods

Two questionnaires were used for data collection to verify the research hypothesis, one for subordinates and the other for leaders. The subordinates evaluated BSL of their direct manager and their PsyCap and job crafting. The job performance of subordinates was evaluated by their direct manager. This was done to secure the objective measurement of job performance and to prevent the common method bias [65]. As subordinates may feel burdened in assessing their current immediate manager, the authors directly explained the purpose of this research and the confidentiality of responses. Moreover, the manager’s survey was conducted two months after the subordinates’ survey to block a potential interaction effect. All questions in the survey used a 7-point Likert scale. This scale has the advantage of drawing more accurate values due to less centralized responses and slightly increased sensitivity of each item [66].

The survey was conducted from April to July 2021 among office workers in 11 companies covering several industries, including manufacturing, construction, IT, and biotechnology in South Korea. A total of 428 questionnaires were distributed; 261 were collected for a collection rate of 61%. Among the collected questionnaires, those with missing values, insincere responses, and mismatches between leader and subordinate were excluded, and a total of 238 were used for the final analysis. The demographic distribution of the collected data by gender was 182 males (76.5%) and 56 females (23.5%). The average age was 35.3 years (SD 6.37). By industry, there were 101 respondents in manufacturing, 78 in construction, and 59 in other industries, such as IT and biotechnology.

The questionnaire was designed as follows. To measure BSL, a tool developed by Faraj and Yan [2] was used. BSL is a comprehensive concept representing the type of behavior that searches for extra-organizational information and resources necessary for business performance. The questionnaire consists of four items including “To what extent does my leader value team members for making use of their relationships with others on behalf of the team”?

For job crafting, 21 items divided into four dimensions developed by Tims, Bakker, and Derks [30] were used. Each of the dimensions of decreasing hindering job demands (e.g., emotionally demanding interactions with others) had six items, and the dimension of increasing structural job resources (e.g., opportunity for development and autonomy), increasing social job resources (e.g., feedback and social support), and increasing challenging job demands (e.g., responsibility and workload) consisted of five items.

To measure PsyCap, 24 items of the Psychological Capital Questionnaire developed by Luthans et al. [67] were used. This tool consists of a total of 24 items divided into six items for each of the four sub-factors of PsyCap: self-efficacy, hope, resilience, and optimism. It includes items such as “I feel confident analyzing a long-term problem to find a solution (self-efficacy)”, “I can think of many ways to reach my current work goals (hope)”, “I usually take stressful things at work in stride (resilience)”, and “When things are uncertain for me at work, I usually expect the best (optimism)”.

Subordinates’ job performance evaluated by the manager was measured by seven in-role behaviors developed by Williams and Anderson [68]. The roles formally required of subordinates by an organization were consisting of seven items, such as “This employee adequately completes the assigned duties”. In addition, in this study, the age and gender of the subordinates and industry type were controlled to clarify the relationship between the variables presented in the research model.

To verify the reliability and validity of the measurement tool before testing specific hypotheses, this study performed confirmatory factor analysis for major variables. The goodness of fit index of the 4-factor model composed of the core factors of this study was found to be at an acceptable level (χ² = 1528.76, CFI = 0.91, TLI = 0.90, RMSEA = 0.02). The index considered to be acceptable when RMSEA is less than 0.08 and TLI and CFI are more than 0.90. Regarding the latent variables, convergent validity was confirmed by calculating Average Variance Extracted (AVE) and Composite Reliability (CR), as shown in Table 1. All of the criteria (AVE > 0.5 and CR > 0.7) suggested by Bagozzi and Yi [69] were satisfied. Dijkstra–Henseler’s ρ_A_ and Cronbach’s α were all confirmed to be more than 0.7 [70,71]. Accordingly, it was determined that there is no problem with the reliability of the data. Because the square root of AVE was larger than correlation coefficient in all cases as shown in Table 2, it was confirmed that data satisfying discriminant validity were obtained [72].

## 4. Results

The descriptive statistics of major variables are summarized as in Table 2. Job performance had a positive correlation with job crafting (*r* = 0.63, *p* < 0.01) and BSL (*r* = 0.39, *p* < 0.01). These results support the nomological consistency in the hypothesis of the mediating effect of job crafting. The relationship between job crafting and PsyCap (*r* = 0.59, *p* < 0.01) also was significant.

**Figure 2:**
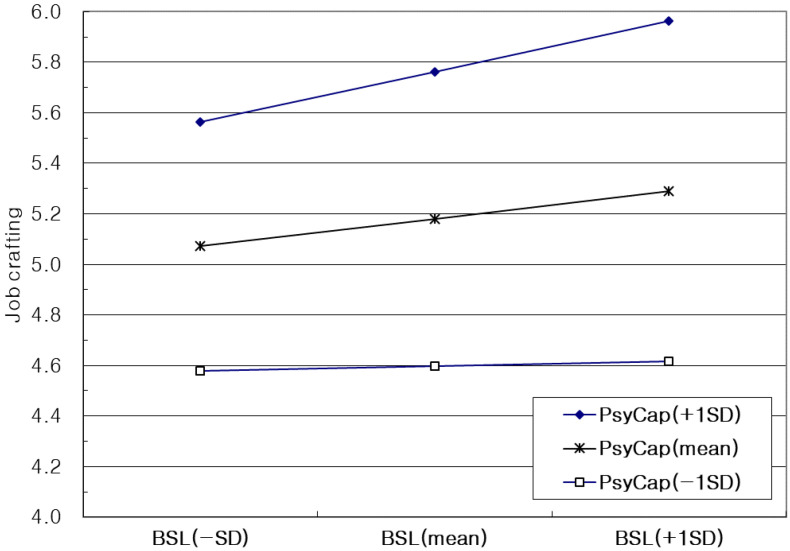
Moderating effect of PsyCap on the relationship between BSL and job crafting.

The results of multiple regression analysis are shown in Table 3. The results of multiple regression of job crafting as a dependent variable are presented in Model 1. Age (*b* = −0.02, *p* < 0.05) and gender (*b* = −0.42, *p* < 0.01) among control variables had a significant effect on job crafting. The effect of BSL (*b* = 0.12, *p* < 0.05) and PsyCap (*b* = 0.64, *p* < 0.01) on job crafting was significant. The product term of BSL and PsyCap was significant (*b* = 0.11, *p* < 0.05), so the effect of BSL on job crafting was confirmed to be positively moderated by PsyCap. This term was generated by the mean centering method to prevent multicollinearity problems. Figure 2, which summarizes simple slope analysis graphically, displays the conditional effect of PsyCap on the relationship between BSL and job crafting. When PsyCap is low (−1 *SD* from the mean), there is no significant relationship between BSL and job crafting. Job crafting significantly increases when BSL increases for the mean value of PsyCap, and this increase is much sharper for high value (+1 *SD* from the mean) of PsyCap. In Model 2, the regression coefficients of job performance on age (*b* = 0.01, *p* < 0.05) and gender (*b* = 0.05, *p* < 0.05) were not significant. The effect of BSL on job performance was not significant (*b* = 0.10, *p* > 0.05) but job crafting was significant (*b* = 0.41, *p* < 0.01).

We also performed bootstrapping 5000 times to examine the moderating impact of PsyCap on the mediating effect of job crafting. This moderated mediating effect can be precisely tested through the bootstrap procedure using PROCESS macro [73]. The indirect effect from BSL to job performance via job crafting was significant and positive (effect = 0.051, SE = 0.03, 95% CI = [0.002, 0.102]). Moreover, the moderated mediating effect of PsyCap was significant (effect = 0.046, SE = 0.02, 95% CI = [0.006, 0.103]). This result implies that the indirect effect of BSL on job performance through job crafting increases with PsyCap, which is a conditional factor.

## 5. Discussion

This study verified the effect of a managers’ BSL on the job crafting and job performance of their subordinates. First of all, as predicted by the research model, the mediating effect of job crafting between BSL and job performance was found to be significant. We interpreted it as the result of improved performance after subordinates who received external resource and procurement support through BSL conducted job crafting. BSL provides an opportunity to reconsider the meaning and influence of subordinates’ work environment or work itself through the provision of richer resources and connection with the external environment [5,21]. In addition, to build new resources by reinvesting slack resources, subordinates can perform more extra-role behaviors, such as job crafting. So far, the studies verifying the relationship between BSL and job crafting of subordinates have been insufficient, but this study extended the claims for the influence of managers’ BSL.

Second, the effect of manager’s BSL on job crafting of subordinates was moderated by PsyCap. As PsyCap was higher, the positive relationship between the manager’s boundary-spanning behaviors and job crafting of subordinates was stronger. This result is in line with the resource conservation theory [51,52], which claims that psychological resources, such as positive emotions, motivation, and energy help employees to focus more on their jobs and to be more active. This suggests that PsyCap makes the subordinates actively engage in job crafting by enabling them to positively recognize the inflow of new information through BSL and contributes to accepting and absorbing that information.

### 5.1. Theoretical Implications and Practical Implications

The theoretical implications in these results are summarized as follows. First, this study helped to understand the meaning and influence of BSL. In the field of traditional leadership research, studies have mainly conducted from internally oriented perspectives, such as how to control and manage subordinates and how to solve problems occurring within an organization [3,7]. However, this study demonstrated the externally oriented role of a manager as a boundary-spanning actor. The results uncovered the black box, the effectiveness creation process of BSL, which has been pointed out as a limitation in previous studies of BSL. Although the relationship between BSL and exploratory activities, such as creativity, has been studied in the sense of changing the boundaries [5], our result shows that BSL also has a positive effect on the prescribed job performance.

Second, this study demonstrated that the leadership, if focused on BSL, can play a motivating role and provide various sufficient tangible and intangible resources for subordinates to perform job crafting. So far, research on job crafting has been mainly conducted regarding the person–job fit. In contrast, this study extracted the common characteristic of improving boundaries from BSL and job crafting and examined the relationship between them. We came to know the role of BSL anew as an antecedent to job crafting.

Third, this study enhanced the current theoretical understanding by introducing the relationship between job crafting and job performance [15] as well as newly adding another rationale based on the effects of BSL. As job crafting encouraged by BSL builds new job resources and pursues a positive self-image by reinvesting existing personal or job resources, it has a positive impact on job performance.

Lastly, PsyCap was demonstrated as having a moderating effect on BSL and job crafting of subordinates under the hypothesis that the effect of a manager’s boundary-spanning behavior could appear different depending on the subordinates’ condition. Our results demonstrated that workers with a high level of PsyCap put more effort and energy into work-related behaviors [16], thus taking initiative, such as job crafting, which resulted in improved job performance.

The practical implications can be summarized as follows. First, we were able to recognize BSL as an effective manager behavioral norm from the consequences regarding its effects on job crafting and job performance of subordinates. Accordingly, the leadership development that encourages not only internal but also external management will be an important way to overcome the business uncertainties. Second, we could find the organizational need for eliciting the positive cognitive and psychological attitudes of subordinates based on the results demonstrating the interaction of the manager’s BSL and subordinates’ PsyCap for job crafting. Therefore, there is a need to consider PsyCap to maximize the potential of subordinates as an organization promotes boundary-spanning management.

### 5.2. Limitations and Future Directions

Despite the above implications and contributions, the following limitations exist in this study. We used two types of questionnaires, which measure independent and dependent variables separately, to avoid the same method bias. However, a limitation remains in that the temporal precedence condition was not satisfied for establishing complete causality. Accordingly, in future research, the causal relationship among BSL and various job-related behaviors of subordinates will need to be clarified through a multi-wave longitudinal or experimental study. Second, this study was conducted with job performance as a representative outcome variable, but, in practice or theory, there are more dimensions regarding job performance that should be treated as important. The effect of BSL on not only to overall job performance but also to contextual performance and counterproductive work behavior [46] will be an important research area in the future. This research expansion will help in comprehensively understanding the effectiveness of BSL. Third, this study was conducted on Korean companies in various industries, and it is meaningful that the effect of boundary-spanning leadership, which is still lacking in research, was examined targeting these organizations. However, there is a sampling limit in generalizing the research results to other countries. It is necessary to overcome this limitation through follow-up studies and including more countries. Future research overcoming the abovementioned limitations will not only contribute to improve the overall understanding of BSL but also help improve managers’ behavior, which have practical effects.

## 6. Conclusions

This study aims to explain the mechanism by which a manager’s boundary-spanning leadership (BSL) affects the job performance of subordinates. The authors also focus on the mediating effect of job crafting as well as the moderating effect of positive psychological capital (PsyCap) and verify that the effects were significant. Based on the results, BSL should first consider PsyCap when encouraging job crafting in order to boost job performance. We propose the practical need for managers’ boundary-spanning behavior, which builds mutual relationships with other external organizations and takes the lead in disseminating external information and resources within the organization under the higher complex business environment. Furthermore, our findings provide a novel insight that the psychologically healthy subordinates could play a more proactive role in the process of BSL enhancing job crafting.

## Figures and Tables

**Table 1:** Reliability and validity.

Factors	Factor Loadings	AVE	CR	ρ_A_	Cronbach’s α
Boundary spanning leadership	0.581~0.885	0.63	0.72	0.75	0.73
Positive psychological capital	0.641~0.861	0.71	0.82	0.86	0.83
Job crafting	0.708~0.906	0.73	0.85	0.88	0.85
Job performance	0.718~0.848	0.76	0.81	0.80	0.79

**Table 2:** Descriptive statistics and correlation matrix.

Variables	Mean	SD	1	2	3	4	5	6	7	8
1. Age	35.28	6.37	-							
2. Gender ^a^	0.24	0.43	−0.35	-						
3. Industry1 ^b^	0.33	0.47	0.06	−0.12	-					
4. Industry2 ^c^	0.25	0.43	−0.07	−0.02	0.04	-				
5. BSL	5.58	0.88	0.07	−0.19	0.07	0.18	(0.79)			
6. PsyCap	5.46	0.71	0.22	−0.16	0.01	0.04	0.45	(0.84)		
7. Job crafting	5.06	0.91	0.31	−0.34	0.10	−0.01	0.37	0.59	(0.85)	
8. Job performance	5.18	0.94	0.27	−0.22	0.18	0.09	0.39	0.58	0.63	(0.87)

Notes: n = 238. Correlation coefficients above |0.13| are significant at 0.05 level, and they above |0.17| are significant 0.01 level (2-tailed). ^a^ Female based on male. ^b^ Construction industry based on manufacturing. ^c^ The other industries based on manufacturing. Square root of AVE values are presented in parenthesis. BSL: Boundary-spanning leadership; PsyCap: Positive psychological capital.

**Table 3:** Regression estimates for job crafting and job performance.

Variables	Model 1 (Job Crafting)			Model 2 (Job Performance)		
	b	SE	*t*	b	SE	*t*
Age	0.02	0.01	2.33 *	0.01	0.01	1.61
Gender	−0.42	0.11	−3.70 **	0.05	0.11	0.51
Industry1	0.12	0.10	1.22	0.25	0.10	2.67 **
Industry2	−0.10	0.11	−0.97	0.15	0.13	1.26
Boundary-spanning leadership (BLS)	0.12	0.06	2.02 *	0.10	0.07	1.45
Job crafting				0.41	0.07	5.54 **
Positive psychological capital (PsyCap)	0.64	0.07	8.81 **			
BLS × PsyCap	0.11	0.04	2.52 *			
R^2^ (adjusted R^2^)	0.45(0.43)			0.34(0.31)		
F	27.03 **			16.37 *		

Notes: * *p* < 0.05, ** *p* < 0.01 (2-tailed).

## Data Availability

The data presented in this study are available on request from the corresponding author. The data are not publicly available due to privacy and ethical considerations.
